# Propagation of pathological α-synuclein in marmoset brain

**DOI:** 10.1186/s40478-017-0413-0

**Published:** 2017-02-02

**Authors:** Aki Shimozawa, Maiko Ono, Daisuke Takahara, Airi Tarutani, Sei Imura, Masami Masuda-Suzukake, Makoto Higuchi, Kazuhiko Yanai, Shin-ichi Hisanaga, Masato Hasegawa

**Affiliations:** 1 Department of Dementia and Higher Brain Function, Tokyo Metropolitan Institute of Medical Science, Tokyo, 156-8506 Japan; 2 Department of Biological Science, Tokyo Metropolitan University, Tokyo, 192-0397 Japan; 3 National Institute of Radiological Sciences, National Institutes for Quantum and Radiological Science and Technology, Chiba, 263-8555 Japan; 4 Department of Molecular Neuroimaging, Tohoku University Graduate School of Medicine, Sendai, 980-8575 Japan; 5 Department of Pharmacology, Tohoku University Graduate School of Medicine, Sendai, 980-8575 Japan; 6 Animal Research Division, Tokyo Metropolitan Institute of Medical Science, Tokyo, 156-8506 Japan

**Keywords:** α-synuclein, Parkinson, Prion, Marmoset, Circuits

## Abstract

α-Synuclein is a defining, key component of Lewy bodies and Lewy neurites in Parkinson’s disease (PD) and dementia with Lewy bodies (DLB), as well as glial cytoplasmic inclusions in multiple system atrophy (MSA). The distribution and spreading of these pathologies are closely correlated with disease progression. Recent studies have revealed that intracerebral injection of synthetic α-synuclein fibrils or pathological α-synuclein prepared from DLB or MSA brains into wild-type or transgenic animal brains induced prion-like propagation of phosphorylated α-synuclein pathology. The common marmoset is a very small primate that is expected to be a useful model of human diseases. Here, we show that intracerebral injection of synthetic α-synuclein fibrils into adult wild-type marmoset brains (caudate nucleus and/or putamen) resulted in spreading of abundant α-synuclein pathologies, which were positive for various antibodies to α-synuclein, including phospho Ser129-specific antibody, anti-ubiquitin and anti-p62 antibodies, at three months after injection. Remarkably, robust Lewy body-like inclusions were formed in tyrosine hydroxylase (TH)-positive neurons in these marmosets, strongly suggesting the retrograde spreading of abnormal α-synuclein from striatum to substantia nigra. Moreover, a significant decrease in the numbers of TH-positive neurons was observed in the injection-side of the brain, where α-synuclein inclusions were deposited. Furthermore, most of the α-synuclein inclusions were positive for 1-fluoro-2,5-bis (3-carboxy-4-hydroxystyryl) benzene (FSB) and thioflavin-S, which are dyes widely used to visualize the presence of amyloid. Thus, injection of synthetic α-synuclein fibrils into brains of non-transgenic primates induced PD-like α-synuclein pathologies within only 3 months after injection. Finally, we provide evidence indicating that neurons with abnormal α-synuclein inclusions may be cleared by microglial cells. This is the first marmoset model for α-synuclein propagation. It should be helpful in studies to elucidate mechanisms of disease progression and in development and evaluation of disease-modifying drugs for α-synucleinopathies.

## Introduction

Parkinson’s disease (PD) is the second most common neurodegenerative disease after Alzheimer’s disease, and Lewy bodies (LBs) and Lewy neurites (LNs) are characteristic features of PD. Dementia with Lewy bodies (DLB) is also a progressive neurodegenerative disease characterized by the appearance of LBs and LNs in cortex [17, 22]. The discovery of disease-associated mutation in the α-synuclein gene *SNCA* and subsequent immunostaining studies with antibodies demonstrated that α-synuclein is the major component of LBs and LNs [2, 55, 56]. It is also the major component of glial cytoplasmic inclusions (GCIs) in multiple system atrophy (MSA) [54, 58]. These diseases are collectively referred to as α-synucleinopathies. To date, six missense mutations in the *SNCA* gene and occurrence of gene multiplication have been identified in familial forms of PD and DLB [1, 5, 24, 28, 29, 41, 52, 62]. α-Synuclein is a small protein of 140 amino acids, which is localized in presynaptic termini, and is involved in maintenance of synapses and synaptic plasticity. In PD, DLB, or MSA patients, it is deposited in the brain as a filamentous form with cross-β structure [51], which is abnormally phosphorylated at Ser129 and partially ubiquitinated [15, 21]. α-Synuclein is natively unfolded, but readily assembles into amyloid-like fibrils under appropriate conditions. Pathogenic mutations affect fibril formation in vitro, either accelerating fibril formation [6, 7, 16] or resulting in formation of fibrils that are more fragile and easier to propagate than wild-type (WT) fibrils [61]. Moreover, the spreading of pathological α-synuclein is closely correlated with disease progression; indeed, the distribution pattern and spread of the pathologies are useful for disease staging of sporadic PD [3, 48]. These results suggest that intracellular amyloid-like α-synuclein fibrils can cause PD and DLB, and spreading of α-synuclein pathology in the brain is considered to be the underlying mechanism of progression of these diseases. Recently, it was experimentally demonstrated that intracerebral injection of synthetic α-synuclein fibrils and/or insoluble α-synuclein from diseased brain converts normal α-synuclein into an abnormal form, and the abnormal α-synuclein propagates throughout the brain in a prion-like manner in WT mouse [30, 33, 34, 57], α-synuclein transgenic mouse [31, 36, 60] and monkey [44].

Common marmoset (*Callithrix jacchus*) is a very small new world primate, about 25 – 35 cm in height and 300 – 500 g in weight, and is far more experimentally tractable than macaque monkey. Since it has high fecundity, with a short sexual maturation period of 18 months, it is attracting increasing attention as an experimental model of primates. In fact, a national project called Brain/MINDS (Brain Mapping by Integrated Neurotechnologies for Disease Studies) was started in 2014 in Japan to develop the common marmoset as a model animal for neuroscience [19, 38, 39]. The marmoset cortex is relatively smooth, but the gyrencephalic and cortical sheet is divided into functionally distinct cortical areas, as in Old World monkeys [45], and thus is suitable for studies of higher cognitive functions and social communication [11]. Therefore, marmosets are considered to be a good experimental model animal to understand the evolution of brain development and function. Moreover, transgenic marmosets have already been generated, demonstrating the feasibility of gene manipulation in this species [49].

To date, mouse models have been used to investigate brain development, circuits, and higher cognitive functions, but they have limitations for exploration of the evolution and development of the primate neocortex. In situ hybridization analysis of marmoset brain revealed that the expression patterns of the genes that regulate brain development (such as EphA6) are different, especially in brain areas that have connections to the prefrontal cortex and are presumably involved in higher cognitive functions, although similar broad regional patterns of expression were observed in both species [32].

A particular difference in brain development and structure between mouse and marmoset is that striatum of marmoset is separated into caudate nucleus and putamen, while these are not distinguishable in rodents. It has been considered that caudate nucleus and putamen were originally one structure and that they became separated by the internal capsule during evolution [25]. Thus, the marmoset has advantageous characteristics as an experimental animal to study brain networks, functions and disease conditions.

Here, we investigated whether intracerebral injection of α-synuclein fibrils can induce PD/DLB-like pathologies in marmoset, and we present the first marmoset model of α-synuclein propagation. We found that marmosets developed abundant phosphorylated α-synuclein pathologies, similar to those observed in PD/DLB, in various brain regions, including striatum, cortex and substantia nigra, at only three months after injection. Remarkably, many LB-like inclusions are observed in tyrosine hydroxylase (TH)-positive dopamine neurons, and a significant decrease in TH-staining was seen in the injection hemisphere. The inclusions were also positive for fluorescent β-sheet ligands, thioflavin-S and FSB, implying that α-synuclein deposits in these animals should be detectable in vivo by positron emission tomography (PET) with a suitable small-molecular agent. Taking account of the advantages of marmosets over mice, we believe the current experimental model would be particularly useful to examine the relationships between PET-detectable α-synuclein lesions and disruptions of neural networks in the absence and presence of candidate α-synucleinopathy-modifying therapeutics.

## Materials and methods

### Preparation of recombinant α-synuclein and fibrils

Recombinant human and mouse wild-type α-synuclein and fibrils were prepared as described previously [33, 57]. Briefly, purified α-synuclein (7 – 10 mg/ml) was incubated at 37 °C in a shaking incubator at 200 rpm in 30 mM Tris–HCl, pH 7.5, containing 0.1% NaN_3_, for 72 h. α-Synuclein fibrils were pelleted by spinning the assembly mixtures at 113,000 xg for 20 min, resuspended in 30 mM Tris–HCl buffer (pH 7.5), and sonicated for 3 min (Biomic 7040 Ultrasonic Processor, Seiko). The protein concentrations were determined by HPLC. Samples were run on gradient 12% polyacrylamide gels and stained with Coomassie Brilliant Blue (CBB), or electrophoretically transferred to PVDF membranes. For immunoblotting, membranes were incubated with 3% gelatin (Wako) for 10 min at 37 °C, followed by overnight incubation at room temperature with primary antibodies. Next, the membranes were incubated for 1 hr at room temperature with biotinylated anti-rabbit or mouse IgG (Vector Lab), then incubated for 30 min with avidin-horseradish peroxidase (Vector Lab), and the reaction product was visualized by using 0.1% 3,3-diaminobenzidine (DAB) and 0.2 mg/ml NiCl_2_ as the chromogen. For electron microscopy, samples were placed on collodion-coated 300-mesh copper grids, stained with 2% (v/v) phosphotungstate, and examined with a JEOL 1200EX electron microscope.

### Marmosets

According to animal protection considerations based on the 3R (reduce, reuse, recycle) principle, we designed the experiment very carefully to minimize the number of animals used. Two female 26-month-old marmosets (individual recognition No. 14H and 14I; born on 4th April, 2014 and bred at the Animal Research Division, Tokyo Metropolitan Institute of Medical Science) were used for this experiment.

### Stereotaxic surgery

The marmosets were anesthetized with Ketamine Hydrochloride (20–40 mg/kg i.m.) and Xylazine (0.05 mg/kg i.m.), and Butorphanol (0.05–0.1 mg/kg i.m.). Then, 50 μL aliquots of 4 mg/mL mouse α-synuclein fibrils were injected into both caudate nucleus (interaural +9.5 mm, Lateral 3 mm, Depth 6 mm) and putamen (interaural +9.5 mm, Lateral 6 mm, Depth 3 mm) in the right hemisphere of 14H brain (total 400 μg). A 50 μL aliquot was injected into caudate nucleus (interaural +9.5 mm, Lateral 3 mm, Depth 6 mm) in the right hemisphere of 14I brain (total 200 μg). The marmosets were bred for 3 months after injection in a biological safety level 2 (BSL-2) environment. All experimental protocols were approved by the Animal Care and Use Committee of Tokyo Metropolitan Institute of Medical Science (No. 16038).

### Antibodies

Primary antibodies used in this study are listed in Table 1. An anti-phosphorylated α-synuclein rabbit monoclonal antibody to pS129 (Abcam) and other anti-α-synuclein antibodies, including LB509 [26] (a gift from Dr Iwatsubo), 75–91 (Cosmo bio), 131–140 (Cosmo bio) and #2642 (Cell Signaling Technology) were used for detection and characterization of α-synuclein pathologies in marmoset brains. Anti-p62 (Progen), anti-Ub (Dako, Millipore), anti-TH (Millipore), anti-NeuN (Millipore), anti-GFAP (Sigma), anti-CNPase (Abcam) and anti-Iba1 (Wako) antibodies were also used.

**Table 1:** Antibodies used in this study

Primary antibodies	Type	Source	Dilution
pS129 (phosphorylated a-syn)	rabbit mono	Abcam (ab51253)	1:2000
LB509 (human a-syn)	mouse mono	Gift from Dr Iwatsubo	1:1000
75–91 (a-syn 75–91)	rabbit poly	Cosmo bio (CAC-TIPSNP08)	1:1000
131–141 (a-syn 131–140)	rabbit poly	Cosmo bio (CAC-TIPSNP09)	1:1000
#2642 (a-syn)	rabbit poly	Cell Signaling Tech (#2642)	1:1000
Anti-p62	guinea pig poly	Progen (GP62-C)	1:1000
Anti-Ub	rabbit poly	Dako (Z0458)	1:1000
Anti-Ub	mouse mono	Millipore (MAB1510)	1:1000
Anti-TH	rabbit poly	Millipore (AB152)	1:1000
Anti-TH	mouse mono	Millipore (MAB318)	1:1000
Anti-NeuN	mouse mono	Millipore (MAB377)	1:1000
Anti-GFAP	mouse mono	Sigma (G3893)	1:1000
Anti-CNPase	mouse mono	Abcam (ab6319)	1:200
Anti-Iba1	rabbit poly	Wako (016–20001)	1:1000

### Immunohistochemistry

Marmosets were deeply anesthetized with pentobarbital injection and killed, and the brain was perfused with 0.1 M phosphate buffer, followed by 10% formalin neutral buffer solution. After fixation, whole brains were sectioned coronally at 50 μm using a vibratome (Leica, Wetzlar, Germany). For high-sensitivity detection, free-floating brain sections were treated with formic acid for 20 min, washed, and boiled at 100 °C for 20 min as described [33]. Sections were then incubated with 0.5% H_2_O_2_ in methanol for 30 min to inactivate endogenous peroxidases, blocked with 10% calf serum in PBS for 20 min, and incubated overnight with appropriate antibodies. After incubation with the biotinylated secondary antibody for 2 h, labeling was detected using the ABC staining kit (Vector) with DAB. Sections were counterstained with hematoxylin. Slides were coverslipped with mounting medium. Images were observed with an all-in-one microscope/digital camera (BZ-X710; Keyence).

For double-label immunofluorescence detection, brain sections were pretreated as described above and incubated overnight at 4 °C with a cocktail of appropriate primary antibodies. The sections were washed and incubated with a cocktail of Alexa568-conjugated goat anti-mouse or anti-rabbit IgG and Alexa488-conjugated goat anti-mouse or anti-rabbit or anti-guinea pig IgG (Molecular Probes). After further washing, the sections were coverslipped with non-fluorescent mounting media (VECTASHIELD; Vector Laboratories) and observed with the BZ-X710.

To measure positive cells, 7–9 sections of substantia nigra were randomly selected, and all images were captured with BZ-X710 microscope using the same settings. The areas of pS129-positive cells and TH-positive cells in the right and left substantia nigra were extracted and quantified by BZ-H3C Hybrid Cell Count Software (Keyence).

### Thioflavin-S and FSB stainings

Thioflavin-S and FSB [23, 50] were purchased from Sigma-Algrich and Dojindo, respectively. For fluorescence labeling with β-sheet ligands, thioflavin-S and FSB, brain sections were mounted on a glass slide and dried with warm air. Sections were incubated in 20% ethanol containing 0.001% β-sheet ligands at room temperature for 30 min. The samples were rinsed with 20% ethanol for 5 min, dipped into distilled water twice for 3 min each, and mounted in VECTASHIELD. Fluorescence images were captured using a BZ-X710 fluorescence microscope equipped with Filter set ET-ECFP (Chroma Technology) for thioflavin-S and FSB. After fluorescence microscopy, all sections labeled with ligands were autoclaved for antigen retrieval, immunostained with the pS129 antibody, and examined using the BZ-X710 instrument.

## Results

### Inoculation of mouse α-synuclein fibrils into marmoset brain

**Fig. 1:**
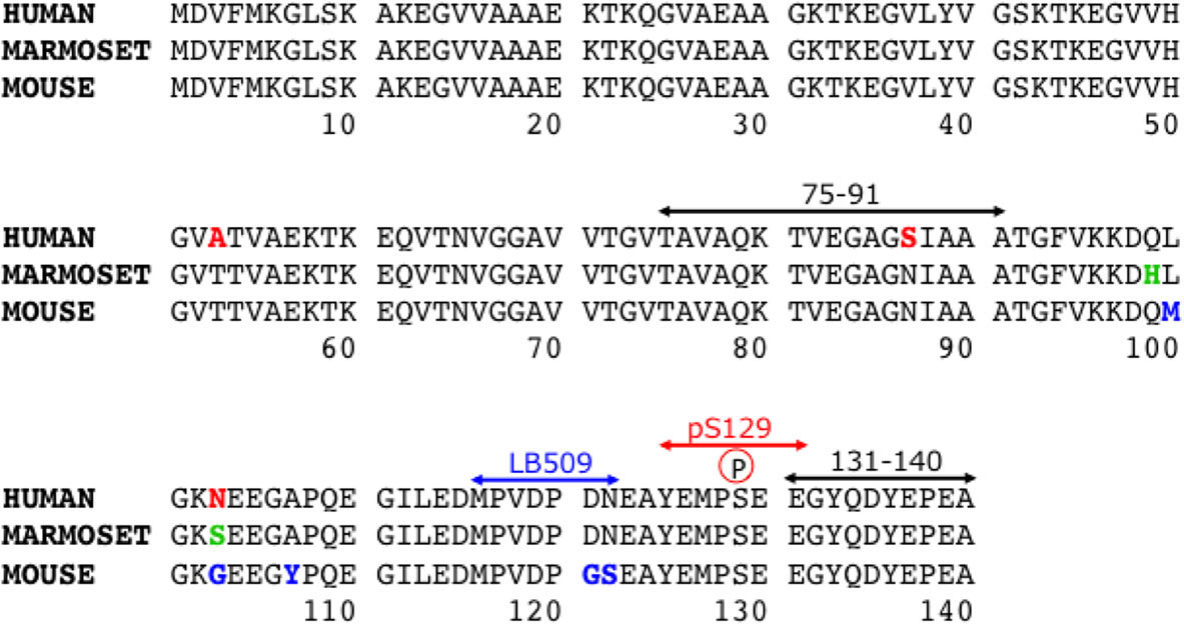
Comparison of amino acid sequences of human (*Homo sapiens*), marmoset (*Callithrix jacchus*) and mouse (*Mus musculus*) α-synuclein. Amino acids in human α-synuclein that differ from those of marmoset and mouse α-synuclein are indicated in red, while amino acids in marmoset and mouse α-synuclein that differ from those in human α-synuclein are indicated in green and blue, respectively. Epitopes of α-synuclein antibodies used in this study are also indicated

**Fig. 2:**
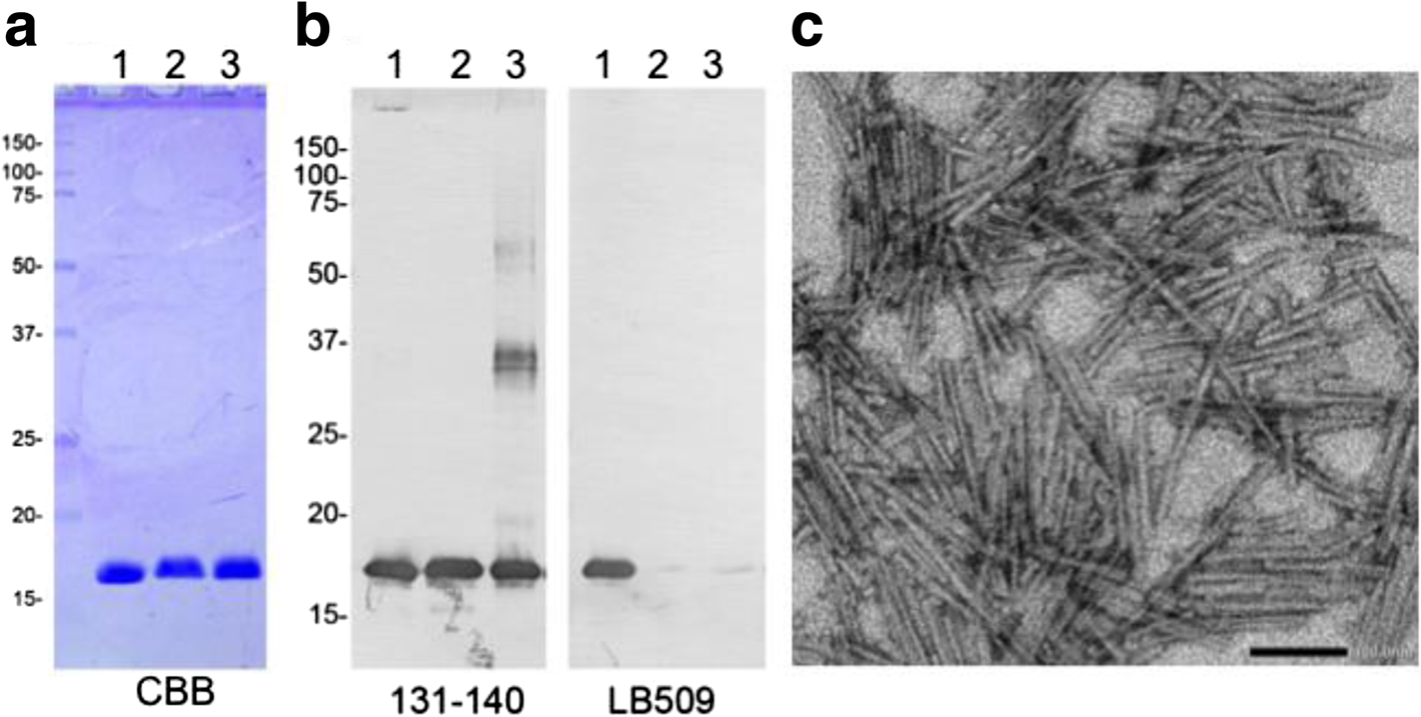
Characterization of purified mouse α-synuclein and fibrils. **a**, purified monomeric human α-synuclein (lane 1), mouse α-synuclein (lane 2) and mouse α-synuclein fibril (lane 3) samples were analyzed by CBB staining (0.5 μg of protein/lane). **b**, results of immunoblotting with anti-α-synuclein antibodies (131–140 and LB509) (B, 0.01 μg of protein/lane). **c**, electron microscopy of α-synuclein fibrils injected into marmoset brains in this study. Negatively stained short fibrils, 50 –300 nm in length, were observed. Scale bar: 100 nm

Marmoset α-synuclein, mouse α-synuclein and human α-synuclein share 96 – 97% amino acid sequence homology (Fig 1), but marmoset and mouse α-synuclein proteins both have a threonine residue at amino acid position 53, which is an aggregation-prone mutation in familial Parkinson’s disease [41]. Therefore, we used mouse α-synuclein fibrils instead of human α-synuclein fibrils for injection into marmoset brains in this experiment. This had the advantage that we could investigate whether endogenous marmoset α-synuclein is deposited in brains injected with mouse α-synuclein fibrils, because the two can be distinguished with antibodies such as LB509. Recombinant mouse α-synuclein and the fibrils were prepared as described, and images of purified recombinant mouse α-synuclein and the fibrils are shown in Fig 2. EM pictures of the injected sample showed that most of the fibrils were straight, 5 – 10 nm in width, and 50 – 300 nm in length (Fig 2C). The prion-like seeding activity of the fibrils to convert normal α-synuclein into abnormal form was checked in our cultured cell model [37] and mouse model [57] (data not shown). We injected 200– 400 μg of the α-synuclein fibrils; this amount was chosen based on the amount used in the previous mouse experiments (10 μg fibrils/0.3 g mouse brain vs 200 μg fibrils/7 g marmoset brain).

### PD-like pathologies in marmoset brain within only 3 months after injection

**Fig. 3:**
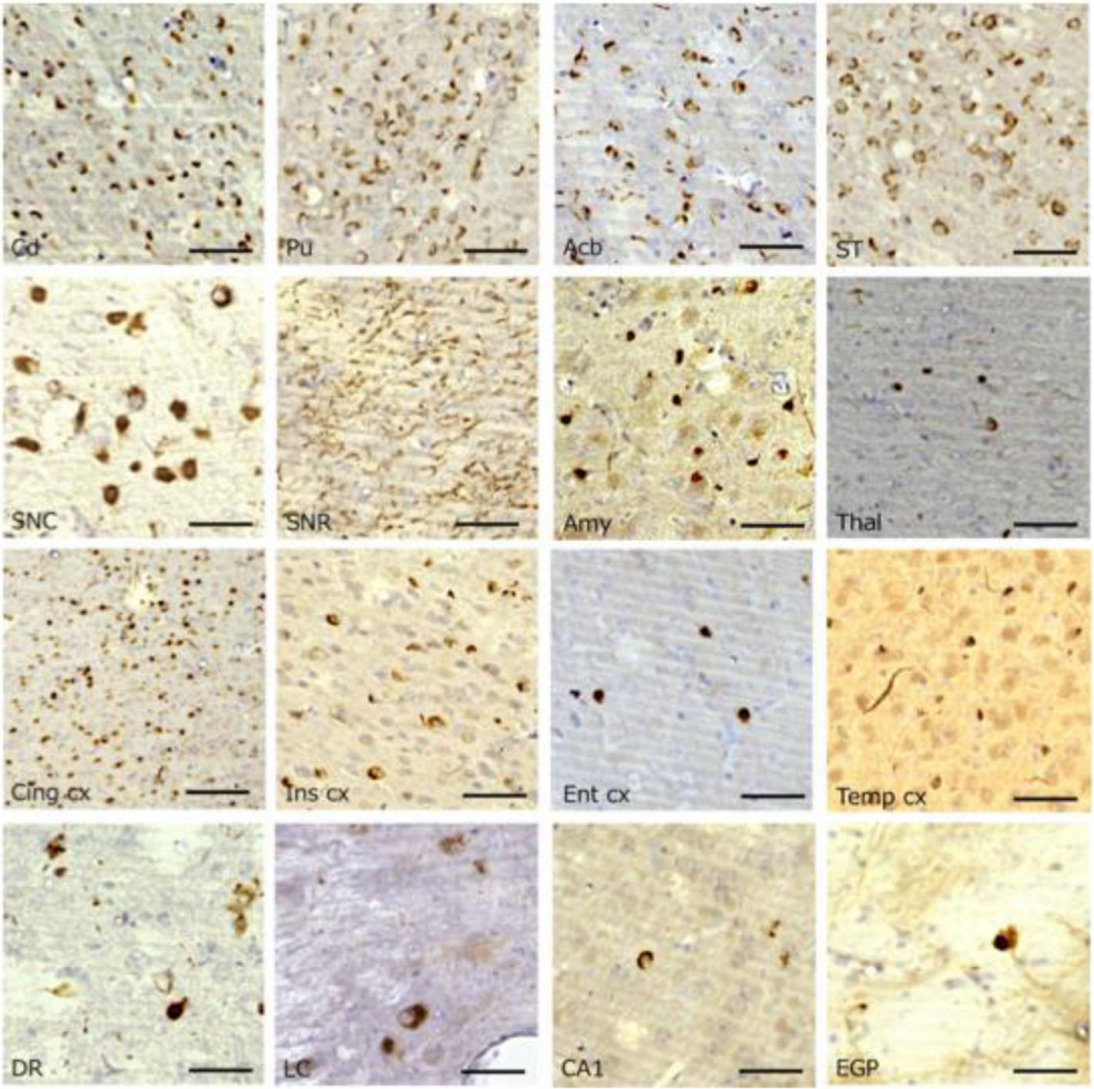
Immunostainings of 14H brain sections with anti-pS129 antibody. PS129-positive inclusions observed in various brain regions. Cd: caudate nucleus, Pu: putamen, Acb: accumbens, ST: bed nucleus of the stria terminal, SNC: substantia nigra compacta, SNR: substantia nigra pars reticulata, Amy: amygdala, Thal: thalamus, Cing cx: cingulate cortex, Ins cx: insular cortex, Ent cx: entorhinal cortex, Temp cx: temporal cortex, DR: dorsal raphe nuclei, LC: locus ceruleus, CA1: hippocampal CA1, EGP: external segment of globus pallidus. Similar pS129-positive inclusions were also observed in 14I marmoset brain. Scale bars: 50 μm

**Fig. 4:**
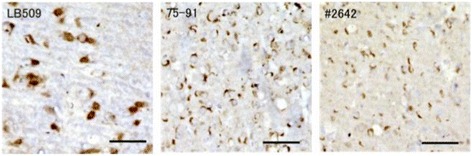
Immunostainings of 14H brain sections with anti-α-synuclein antibodies (LB509-positive inclusions in substantia nigra and stainings with 75–91 and #2642 in caudate nucleus). Similar inclusions were observed in 14I marmoset brain. Scale bars: 50 μm

**Fig. 5:**
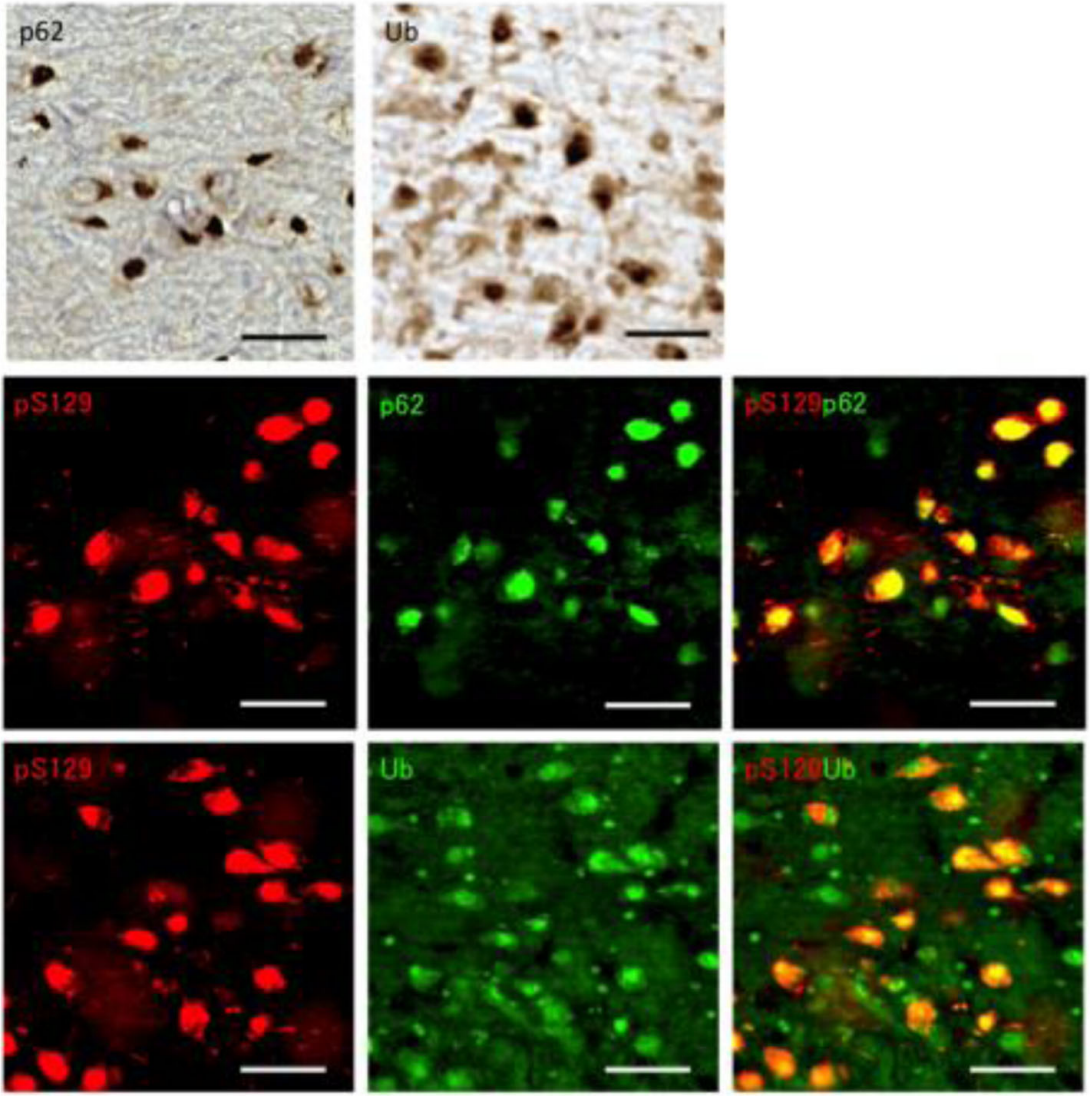
Immunostainings of 14H brain sections (substantia nigra) with anti-p62 and anti-ubiquitin (anti-Ub) antibodies. Diaminobenzidine staining (upper panel) and double immunofluorescence stainings (middle and lower panel) indicated colocalization of pS129-positive inclusions and p62 or ubiquitin. Similar results were obtained in other brain regions and also in 14I marmoset brain. Scale bars: 50 μm

Three months after injection, formalin-fixed marmoset brain sections were prepared and immunostained with anti-phospho-Ser129 of α-synuclein (pS129). Surprisingly, abundant pS129-positive structures were observed throughout the brain regions, including the injection sites in the caudate nucleus and putamen, accumbens, bed nucleus of the stria terminalis, substantia nigra, amygdala, a wide region of cortex, thalamus, globus pallidus, dorsal raphe nuclei, raphe nucleus and hippocampal CA1 in both marmosets (Fig 3). These abnormal α-synuclein pathologies were also positive with other anti-α-synuclein antibodies (Fig 4), including LB509. The results indicated that endogenous marmoset α-synuclein is converted into an abnormal form by inoculation of mouse α-synuclein fibrils and deposited in the brain. Most of the inclusions were also positive for antibodies to ubiquitin and p62 (Fig 5), as is the case in the brains of patients. Labeling of the inclusions with these antibodies was observed without pretreatment of sections with formic acid and boiling, but the specificity and sensitivity were both increased by the pretreatment (data not shown).

### α-Synuclein propagated retrogradely through neuronal networks

**Fig. 6:**
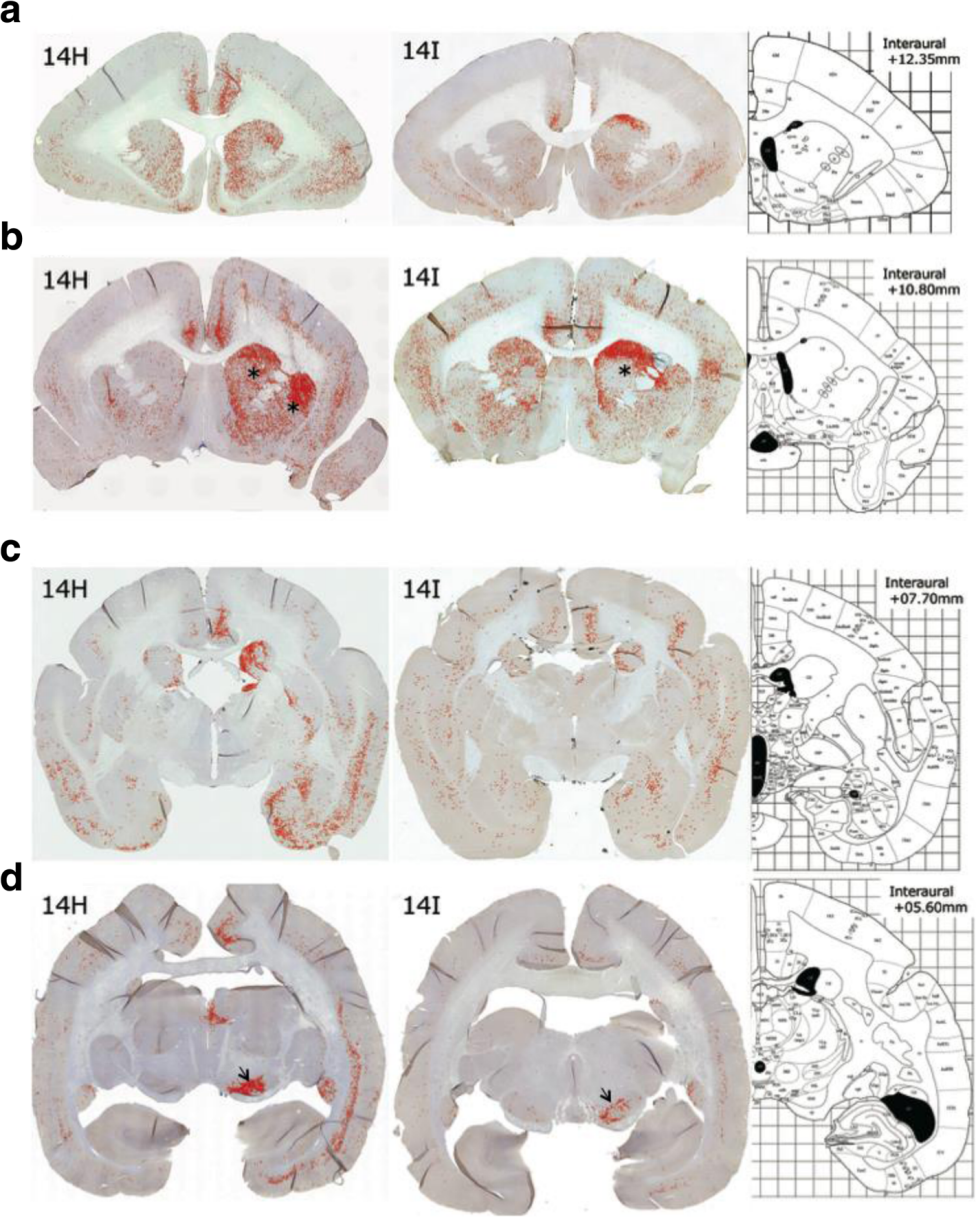
Distribution of pS129-positive α-synuclein pathologies at 3 months after mouse α-synuclein fibril injection in the two marmosets (14H and 14I). The pS129-positive phase contrast images of four coronal brain sections (**a** – **d**) acquired by using BZ-X710 microscope system are shown in red with marmoset brain sections of corresponding brain regions. Coronal brain atlas (Hardman and Ashwell, 2012) locations of interaural +12.35 mm (**a**), +10.80 mm (**b**), +07.70 mm (**c**) and +05.60 mm (**d**) are shown. *Asterisks* in **b** indicate the injection sites in caudate nucleus and/or putamen. *Arrows* in **d** indicate substantia nigra

The distributions of the pathologies were similar in these two marmosets 14H and 14I, although more abundant and severe pathologies were observed in the marmoset 14H (Fig 6), in which α-synuclein fibrils were injected at both caudate nucleus and putamen. The α-synuclein pathologies were robust in the injected right hemisphere, but also present in the left hemisphere, although to a much lesser extent than in the right hemisphere (Fig 6). The spreading of α-synuclein pathology observed in the marmosets seemed consistent with propagation through neuronal networks (Brainstem anatomy WIKI, http://brainstemwiki.colorado.edu/doku.php/start). In these marmosets, α-synuclein pathologies were seen from the injection sites (caudate nucleus and putamen) to neocortex, substantia nigra, amygdala, globus pallidus and thalamus, which are brain nuclei or regions providing direct input to striatum. As projections to the striatum, nigrostriatal input, corticostriatal input, thalamostriatal input, and inputs from the external segment of globus pallidus and subthalamic nucleus have been reported [4, 12–14, 20, 40, 53, 59]. Therefore, pathological α-synuclein seemed to have spread to the second connection regions from the direct input regions over the 3-month period. Overall, the spreading pattern of pathological α-synuclein seemed to be the same as in mouse brain [30, 33, 57], and was consistent with the spreading pattern of retrograde tracers [40].

**Fig. 7:**
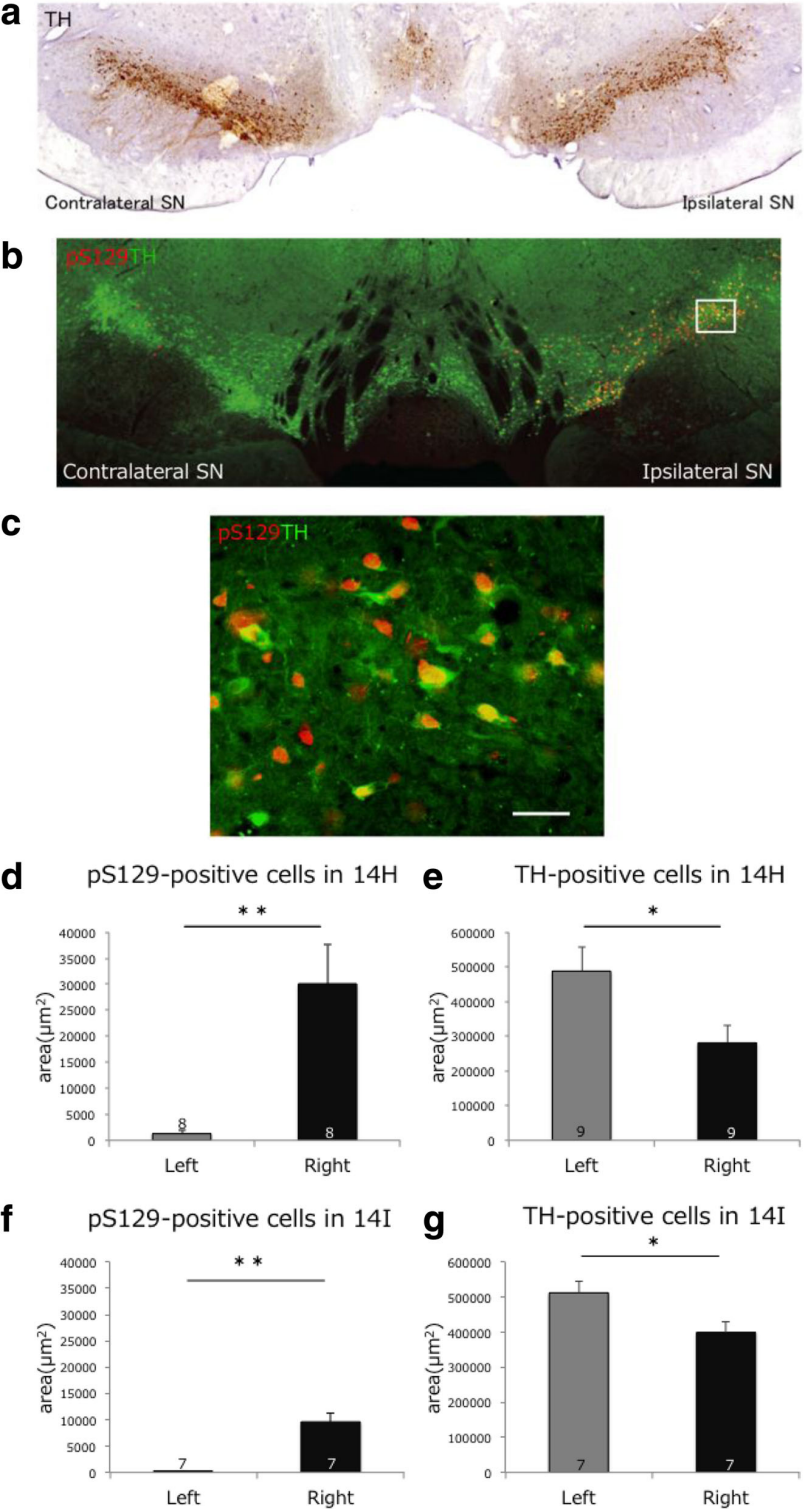
Presence of pS129-positive inclusions in TH-positive neurons and significant reduction of TH-positive neurons in the ipsilateral side of the marmosets (14H and 14I). **a**, Immunohistochemical staining of substantia nigra with anti-TH antibody and diaminobenzidine staining in 14H. **b**, Double-labeling of substantia nigra with anti-TH (green) and anti-pS129 (red) antibodies in 14H. **c**, High magnification of the double-labeling of substantia nigra on the ipsilateral side (indicated by the squares in **b**). An apparent reduction of TH-positive dopamine neurons was detected in the ipsilateral side of the brain compared to the contralateral side. Areas of pS129-positive inclusions and areas of TH-positive neurons were quantified (**d** – **g**). **d**, **e** Quantification of pS129-positive inclusions and TH-positive cells in the right and left hemispheres of 14H brain. **f**, **g** Quantifications of pS129-positive inclusions and TH-positive cells in the right and left hemispheres of the 14I. To measure positive cells, 7– 9 sections (the numbers are indicated in the columns) were selected. Larger amounts of pS129-positive inclusions were detected and significant reductions of TH-positive neurons were detected in the ipsilateral sides of substantia nigra of the two marmosets. Data were analyzed by Student's *t*-test. All error bars indicate means ± S.E.M. **p < 0.002, * *p* < 0.05. Scale bars: 50 μm

It is noteworthy that abundant LB-like round pS129-positive inclusions were detected in substantia nigra of these marmosets (Fig 3, 4, 5 and 7). The nigral LB-like inclusions were more prominent in the marmoset injected into both caudate nucleus and putamen than in the marmoset injected only into caudate nucleus. Double labeling of the inclusions with anti-tyrosine-hydroxylase (TH) antibody confirmed that the inclusions are formed in TH-positive dopaminergic neurons (Fig 7b, c), indicating that pathological α-synuclein was propagated retrogradely from striatum to nigral neurons.

### Degeneration of dopaminergic neurons with pS129-positive inclusions

Macroscopical observation of sections stained with anti-TH antibody indicated an apparent decrease of TH-positive staining in the injected hemisphere (Fig 7a). Therefore, we quantitated TH-positive neurons in the right and left hemispheres and we also quantitated pS129-positive neurons, and compared them. As shown in Fig 7d – e, pS129-positive aggregates were much more abundant in the right hemisphere than in the left hemisphere. On the other hand, a significant decrease in the amount of TH-positive neurons was detected in the right hemisphere in both marmosets, although the reduction was more prominent in the marmoset that developed more pS129-positive aggregates following injection into both caudate nucleus and putamen.

### Most of the aggregates are strongly positive for both FSB and thioflavin-S

**Fig. 8:**
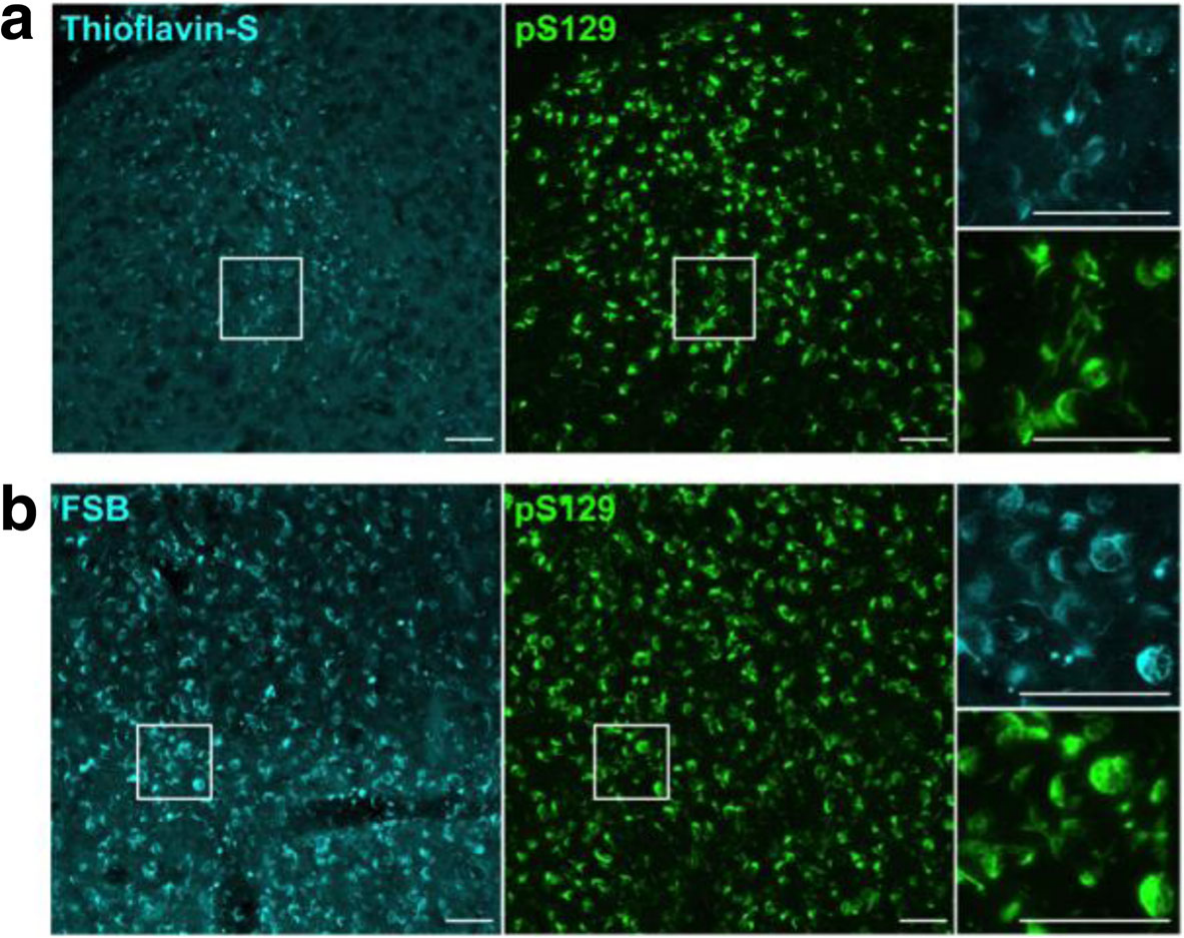
Fluorescence labeling of α-synuclein inclusions with β-sheet ligands in 14H (caudate nucleus). **a** Double staining with 0.001% thioflavin-S (left) and anti-pS129 antibody (middle). Top right and bottom right panels depict high-power photomicrographs of areas indicated by squares in the left and middle panels, respectively. A large proportion of α-synuclein inclusions stained with pS129 was labeled with thioflavin-S. **b** Double staining with 0.001% FSB (left) and pS129 (middle). Top right and bottom right panels depict high-power photomicrographs of areas indicated by the squares in the left and middle panels, respectively. A large proportion of α-synuclein inclusions stained with pS129 was strongly labeled with FSB. Similar labelings were observed in 14I marmoset brain. Scale bars: 50 μm

To further investigate whether the pS129-positive aggregates in these marmosets are amyloid-like structures, we tried to stain them with thioflavin S and FSB. Remarkably, most of these aggregates, including LB-like and LN-like structures, were strongly positive for both thioflavin-S and FSB (Fig 8), indicating that the inclusions formed in marmosets at only 3 months after injection have cross-β structures similar to those found in brains of patients. These results suggest that propagation and spreading of these α-synuclein pathologies in vivo may be monitored by the use of amyloid-imaging probes.

### Degenerating neurons with α-synuclein inclusions may be cleared by microglial cells

**Fig. 9:**
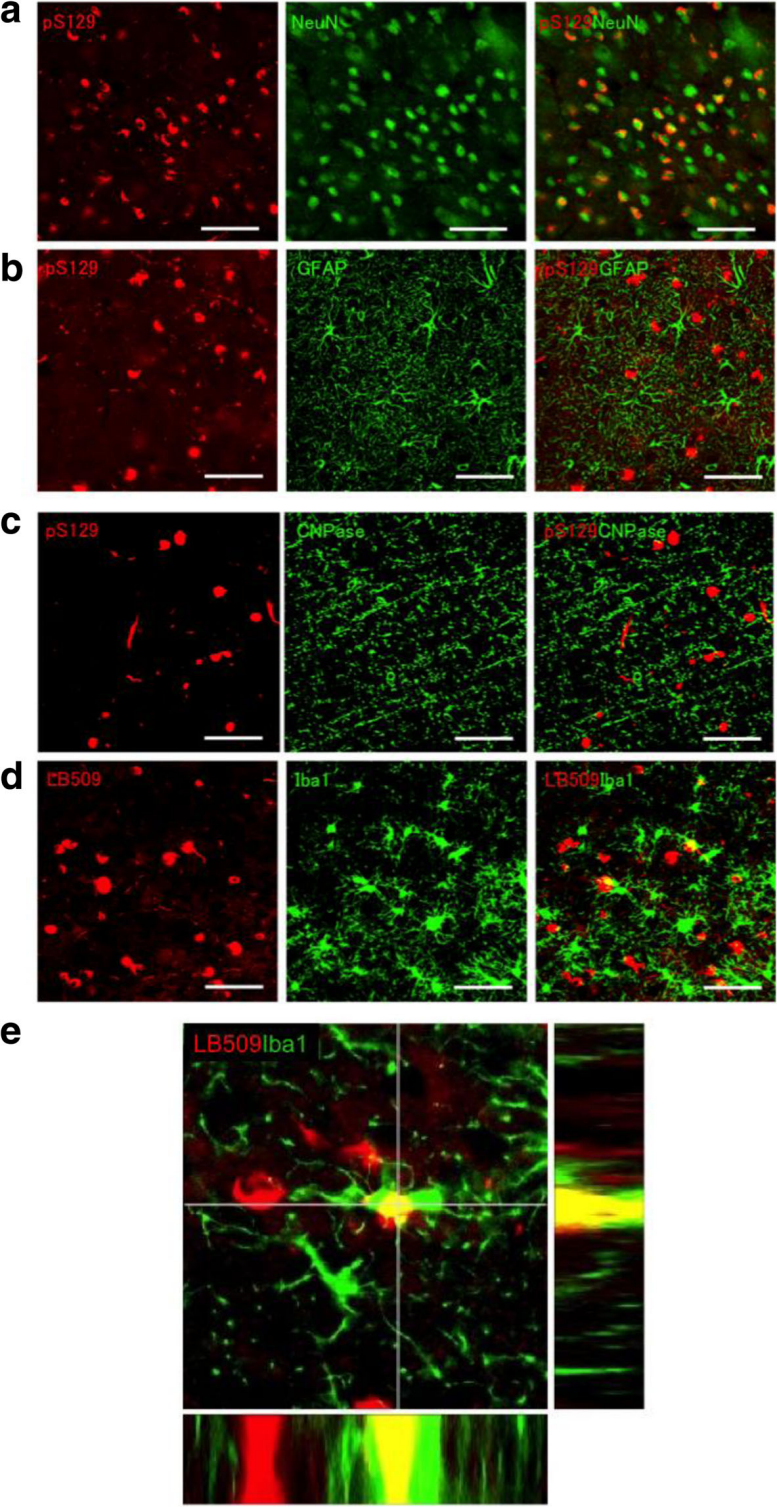
Double immunolabeling of α-synuclein inclusions with pS129 antibody and anti-NeuN, anti-GFAP, anti-CNPase or anti-Iba1 antibodies in 14H (caudate nucleus) **a** Double staining with anti-pS129 and anti-NeuN antibodies. **b** Double staining with anti-pS129 and anti-GFAP antibodies. **c** Double staining with pS129 and anti-CNPase antibodies. **d** Double staining with LB509 and anti-Iba1 antibodies. **e**, A high-power photomicrographs of the double staining with LB509 and anti-Iba1 antibodies. Similar stainings were observed in various other brain regions and also in 14I marmoset brain. Scale bars: 50 μm

All of the inclusions seemed to be neuronal aggregates, and no astrocytic or oligodendrocytic glial inclusions were observed. In fact, most of the pS129-positive inclusions were closely associated with NeuN-positive nuclei in neuronal cells, but no such colocalization was observed with astrocytic marker GFAP or oligodendrocytic marker CNPase (Fig 9a, b and c). Very interestingly, double labeling with LB509 and Iba1 revealed that some of the LB509-positive inclusions were colocalized with Iba1-positive microglial cells (Fig 9d, e), strongly suggesting that the inclusions or degenerating neurons with aggregates may be phagocytosed by microglial cells. The LB509-positive α-synuclein inclusions should be composed of endogenous marmoset α-synuclein, but not injected mouse α-synuclein, since LB509 recognizes human and primate α-synuclein. The colocalization was confirmed by 3D imaging (Fig 9e). Similar colocalization of α-synuclein inclusions and microglial cells was observed by double labeling with anti-pS129 and anti-Iba1 antibodies (data not shown).

## Discussion

Emerging evidence indicates that intracellular amyloid-like proteins have prion-like properties and propagate from cell to cell by converting normal proteins into abnormal forms [18, 22, 27, 42]. This prion-like propagation may account for the characteristic spreading of pathological proteins including α-synuclein, tau and TDP-43, and also for disease progression in major neurodegenerative diseases with these protein pathologies, such as Parkinson’s disease, Alzheimer’s disease and amyotrophic lateral sclerosis.

In this study, we tested whether inoculation of synthetic mouse α-synuclein fibrils can induce PD-like α-synuclein pathologies and prion-like propagation in two adult marmosets by injecting the fibrils into striatum (one animal was injected into caudate nucleus and the other, into caudate nucleus and putamen). Within only 3 months after injection, we observed abundant phospho-α-synuclein pathologies in various brain regions of both marmosets, indicating that prion-like conversion readily occurred in the primate brains even within this short time-scale. Luk et al. and we have established a propagation model in wild-type mouse [30, 33, 34, 57], but others have found it difficult to detect the pathologies in wild-type mouse [46]. It has also been reported that intranigral or intrastriatal inoculations of PD-derived LB extracts in monkey resulted in progressive nigrostriatal neurodegeneration, but clearly defined LB-type inclusions were not observed [44]. The results of the present study clearly demonstrate that inoculation of fibrillar α-synuclein in striatum of wild-type marmoset triggered PD-like α-synuclein pathologies, which propagated retrogradely to substantia nigra and other input regions, and induced degeneration of dopaminergic neurons. Furthermore, most of the inclusions were positive for amyloid-sensitive dyes, such as thioflavin-S and FSB. This simple experiment has provided direct evidence for prion-like propagation of pathological α-synuclein in brains of primates, and the model should be very useful for establishing in vivo imaging methodology for abnormal α-synuclein propagation and for development and evaluation of disease-modifying drugs for α-synucleinopathies.

In this study, we did not perform behavioral tests, but we did not observe any apparent symptoms or behavior deficits in these marmosets, suggesting that they may not develop strong phenotypes within 3 months after inoculation. It is reasonable to speculate that motor deficits would only be detected after the loss of more than 50% of dopamine neurons, as is the case in PD patients. We observed 20 – 40% decrease of TH-positive cells in the right hemisphere in the animals in this study. Further studies will be needed to establish the relationship between pathologies and symptoms in wild-type marmosets.

The present model should be useful for research on PD and α-synucleinopathies, because this is a primate and non-transgenic wild-type animal model, which would not suffer from various artifacts associated with overexpression of proteins in transgenic animals [47] or the use of viral vector-mediated gene transfer systems. Among mouse models, Tg-mice overexpressing human A53T mutant α-synuclein (such as M83 line) are considered a good host animal for inoculation experiments, because disease symptoms and α-synuclein pathologies appear at about ~100 days after inoculation [43]. When Tg-marmoset models overexpressing human α-synuclein are available, it will be interesting to inject synthetic α-synuclein fibrils or brain extracts from patients into these animals to see whether the appearance of PD-like symptoms or pathologies is accelerated.

By double immunolabeling of marmoset brain sections with LB509 and Iba1, we demonstrated that some of the α-synuclein inclusions are colocalized with Iba1-positive microglial cells. This finding suggests that inclusions or degenerating neurons with aggregates may be phagocytosed by microglial cells. Although it has been debated whether inflammation constitutes a cause or consequence of PD, increasing evidence suggests that microglial cells and inflammatory pathways are involved in the pathogenesis and progression of PD [8, 9]. Indeed, activated microglia are prevalent in the most pathologically affected areas in the brains of PD patients [9, 35]. Recent studies also demonstrated that toll-like receptor 2 may contribute to α-synuclein pathology in PD [10]. However, there has been no direct evidence that microglial cells are involved in the clearance of α-synuclein aggregates, and our findings here represent the evidence that α-synuclein aggregates or cells with inclusions are phagocytosed by microglial cells for clearance. It seems plausible that such microglial phagocytosis of α-synuclein inclusions may be a protective event to clear degenerating neurons and reduce inflammation in the brain, but further studies will be needed to confirm this. Our marmoset model should be useful for elucidating the molecular mechanisms of α-synuclein propagation, and also for exploring neuronal circuits in marmoset brain and human brain.

## Conclusions

Intracerebral injection of synthetic α-synuclein fibrils into adult wild-type marmoset brains induced abundant α-synuclein pathologies within only three months after injection. Most of the α-synuclein inclusions were positive for β-sheet ligands (thioflavin-S and FSB). Remarkably, robust Lewy body-like inclusions were formed in TH-positive neurons and a significant decrease in the numbers were observed, strongly suggesting the retrograde spreading of abnormal α-synuclein and the neurotoxicity. Furthermore, we provide evidence indicating that neurons with abnormal α-synuclein inclusions may be cleared by microglial cells. This is the first marmoset model for α-synuclein propagation, and it should be useful for elucidating the molecular mechanisms of α-synuclein propagation, and also for exploring neuronal circuits in marmoset brain and human brain.
